# Knowledge support for environmental information on pharmaceuticals: experiences among Swedish Drug and Therapeutics Committees

**DOI:** 10.1186/s12913-023-09646-7

**Published:** 2023-06-12

**Authors:** Elkanah Linder, Björn Wettermark, Marie-Louise Ovesjö, Sofia Kälvemark Sporrong, Helena Ramström

**Affiliations:** 1 Pharmacoepidemiology & Social Pharmacy, Department of Pharmacy, Uppsala University, Uppsala, Sweden; 2 Pharmacoepidemiology, Department of Pharmacy, Uppsala University, Uppsala, Sweden; 3 Division of Clinical Pharmacology, Department of Laboratory Medicine, Karolinska Institutet, Stockholm, Sweden; 4 Department of Quality and Patient safety, Södersjukhuset, Stockholm, Sweden; 5 Social Pharmacy, Department of Pharmacy, Uppsala University, Uppsala, Sweden; 6 Health and Medical Care Administration, Region Stockholm, Sweden

**Keywords:** Drug and Therapeutics Committees, Environment, Survey, Pharmaceuticals, Knowledge support, Recommended medicines

## Abstract

**Background:**

Two publicly available Swedish knowledge support systems, “Pharmaceuticals and Environment” on Janusinfo.se and Fass.se, provide environmental information on pharmaceuticals. Janusinfo is provided by the public healthcare system in Stockholm and Fass is provided by the pharmaceutical industry. The objectives of this study were to investigate the experiences among Swedish Drug and Therapeutics Committees (DTCs) with using the databases, retrieve development proposals for these, and investigate the DTCs’ challenges with working with pharmaceuticals in the environment.

**Methods:**

A cross-sectional survey with 21 questions, both closed and open-ended, was distributed electronically in March 2022 to Sweden’s 21 DTCs. Descriptive statistics and inductive categorization were used for the analysis.

**Results:**

A total of 132 respondents from 18 regions filled out the survey. The average regional response rate was 42%. The DTCs used the knowledge supports to consider environmental aspects of pharmaceuticals in their formularies and in education. Respondents were more familiar with Janusinfo compared to Fass but appreciated the availability of both. The DTCs especially valued the concrete proposals for certain active pharmaceutical ingredients on Janusinfo. Respondents requested that all medicinal products have environmental information on Fass. Challenges included lack of data, lack of transparency from the pharmaceutical industry and difficulties considering the environmental aspect of pharmaceuticals in their healthcare practice. Respondents wanted more knowledge, clear messages, and legislation to support their work to reduce the negative environmental impact of pharmaceuticals.

**Conclusions:**

This study demonstrates that knowledge supports for environmental information on pharmaceuticals are valuable for the DTCs in Sweden, but the respondents experienced challenges in their work in this field. The study can provide insights to those in other countries interested in considering environmental aspects in their formulary decision-making.

**Supplementary Information:**

The online version contains supplementary material available at 10.1186/s12913-023-09646-7.

## Background

Pharmaceuticals in the environment are more frequently discussed in society as it is understood that most pharmaceutical residues are eventually spread in eco-systems where they could have a negative impact on both human and animal health. Pharmaceutical residues can enter the environment through human consumption and excretion or other pathways, e.g., manufacturing, and as a result, have been detected in environmental matrices, including, but not limited to, surface water, groundwater, drinking water and soil [1–4]. Recently, a global review of pollution in 258 of the world’s rivers, found concentrations of at least one Active Pharmaceutical Ingredient (API) in one-quarter of the sampling sites that were greater than concentrations considered safe for aquatic organisms, or were of concern in terms of selection for antimicrobial resistance [5]. The EU Commission has adopted an action plan towards a toxic-free environment and in the directive 2008/105/EC (under revision) on environmental quality standards in the field of water policy, nine APIs are suggested to be included [6, 7]. For these APIs, emission limit values must not be exceeded for surface water to achieve good chemical status [7].

Inappropriate prescribing was the key driver behind the development of Drug and Therapeutics Committees (DTCs) in the 1960’s [8, 9]. Since then, DTCs have been established in many countries across the world and the World Health Organization (WHO) has produced guidelines on how to establish DTCs as a tool for promoting rational use of medicines [10, 11]. In Sweden, regional DTCs^1^ covering both hospitals and primary care became mandatory in 1997 as part of a national reform to promote a more cost-effective use of medicines in the country [13, 14]. These DTCs promote the rational use of medicines through developing formularies (recommendations for prescribing that are not obligatory to follow), providing continuous professional education and monitoring prescribing patterns. Selection of pharmaceuticals in the regional formularies is primarily based on Evidence-Based Medicine (EBM)-criteria of efficacy and safety, but pharmaceutical suitability and cost-effectiveness are also important criteria [14]. In recent times, the environmental aspect has received more attention and has been included in the formulary for the capital region of Stockholm since 2005 [15–17]. Similar development is seen in other Swedish regions, but to the best of our knowledge, there is yet no published information on similar initiatives from DTCs in other countries.

To make it possible for the DTCs to include the environmental aspect, environmental information for pharmaceuticals needs to be available. In Sweden, a collaboration was initiated in 2001 between Region Stockholm and Apoteket AB (public owner of all pharmacies in Sweden at that time) to provide environmental information on APIs [15, 18]. The characteristics of persistence, bioaccumulation, and ecotoxicity constitute the environmental hazard of pharmaceuticals for human use on the Swedish market. In an extended collaboration with Region Stockholm, Apoteket AB, the Swedish Medical Products Agency, LIF (the trade association for the research-based pharmaceutical industry in Sweden), and the Swedish Association of Local Authorities and Regions, an environmental risk assessment was developed [15, 18]. The risk relates to the likelihood of toxic effects on aquatic organisms, i.e., a comparison between exposure during use and toxicity.

Two publicly accessible knowledge support systems provide environmental information for pharmaceuticals on the Swedish market via their respective websites. The information is available per *API* within the knowledge support “Pharmaceuticals and Environment” page on Janusinfo.se (Janusinfo), provided by Region Stockholm, and per *medicinal product* under the heading “Environmental Information” on Fass.se (Fass), provided by the pharmaceutical industry.

Janusinfo presents environmental information from regulatory authorities and pharmaceutical companies [15]. The knowledge support presents the worst-case scenario of hazard and risk under the summary. Detailed information from the different sources follows and can also include comparative environmental assessments for medically comparable alternatives. In addition, a reference list is presented [15].

On Fass, under the environmental information tab for a medicinal product, environmental risk, degradation, and bioaccumulation are presented [15, 19]. Detailed information is also presented including a reference list. The environmental information is provided by the pharmaceutical companies on a voluntary basis, and information is missing for many medicinal products [15, 20]. In addition, the environmental information can differ between medicinal products with the same API since each pharmaceutical company can provide different environmental information [15, 20].

Currently, other databases are available that provide environmental information on pharmaceuticals with a variety in focus and structure [15]. For example, the WikiPharma database provides ecotoxicity data, and the pharmaceutical companies present information on the properties, environmental fate characteristics, and ecotoxicity of pharmaceuticals in the iPiE (Intelligent Assessment of Pharmaceuticals in the Environment) database. However, none of the available databases directly meet the needs of the DTCs in Sweden [15]. The Norwegian Felleskatalogen (The Norwegian Pharmaceutical Product Compendium) uses environmental information from Fass.se [21]. Finland also uses environmental information for pharmaceuticals from Fass [22]. We have not found any information in the research literature about how the other Nordic countries practically use the environmental information on pharmaceuticals presented in their databases or whether DTCs in other countries consider the environmental aspect in their formularies.

Thus, to our knowledge, there is yet no study of the DTCs’ use of, attitudes regarding and suggestions for knowledge supports for environmental information on pharmaceuticals. Such an analysis is valuable given the growing awareness of the risks posed by pharmaceuticals in the environment and the important role DTCs have in terms of influencing prescribing behavior. Consequently, the aims of this study were to evaluate the attitudes and perceived usefulness of the knowledge supports for environmental information on pharmaceuticals available through Janusinfo and Fass, respectively, among members of Swedish DTCs and to collect their proposals on how to further develop the knowledge supports. Additionally, we aimed to elucidate what they perceive as challenges in their work with environmental aspects of pharmaceuticals.

## Methods

### Study design

A cross-sectional survey was conducted in Swedish including both closed and open-ended questions. The questions were developed based on the research team’s aims and scientific literature [23, 24]. The survey was distributed electronically in March 2022 and followed by three reminders.

### Setting and participant sampling

The target group consisted of chairpersons, selected members of Sweden’s 21 DTCs, as well as a few other environmental coordinators working with pharmaceuticals in the environment on a regional level. Chairpersons were first contacted via email regarding individual and regional participation. The chairpersons then forwarded the email to persons working with the regional formulary and/or with pharmaceuticals in the environment at a regional level. Respondents were included if they were members of or affiliated with the DTC when they got the survey and/or had experience working with the DTC formulary in their region. We applied no specific exclusion criteria.

### Data collection

All data were collected through a web survey that had been pilot tested by 10 respondents having current or previous experience working in a DTC. Pilot test participants were sent a link to the survey to fill out and seven questions to answer about the survey. The seven questions addressed the comprehensibility of the questions; the time it took to complete the survey; thoughts on the multiple choice and open-ended questions; question order; the electronic platform; and any other comments they had. The input from the pilot test participants was used to make changes in the survey including question wording and question structure.

The final survey was distributed in the Uppsala University platform Kurt® to all 21 chairpersons to complete and further distributed. The questionnaire contained 21 questions, some of which were Likert scale alternatives, in five Sects. [24]:

1. background questions (8 items).
2. use of any of the knowledge supports for environmental information on pharmaceuticals (1 item).
3. attitudes to Janusinfo (5 items) (asked to skip if not using).
4. attitudes to Fass (5 items) (asked to skip if not using).
5. other questions about the use of environmental information and challenges working with pharmaceuticals in the environment (2 items).

An additional file [see Supplementary Material 1] shows the survey that was distributed.

### Analyses

The data were analysed using Microsoft Excel® (version 16.16.27). Quantifiable questionnaire data were analysed with traditional descriptive statistics (frequencies, mean and mode). Those who answered that they used either Janusinfo or Fass or both, were included in the analysis of the specific questions about the knowledge supports, the sections above about attitudes to Janusinfo and Fass, respectively. Means were calculated for questions with 3- and 4-point Likert scales by assigning each option a whole number value 1‒3, or 1–4. A value of 1 was associated with the *not at all* or *very difficult* opinions in the Likert scale. A value of 3 or 4, depending on the number of options in the Likert scale (excluding *don’t know*), was associated with the attitudes at the opposite end of the Likert scale, for example, *very important* and *very* easy. The *don’t know* answers were excluded from the mean calculations. For the open-ended questions, quotes were inductively categorised and category counts with representative examples are presented.

## Results

### Background characteristics

A total of 132 respondents filled out the survey. Responses were received from 18 out of 21 regions. The three regions that did not respond were geographically spread and represented 6% of the total population in Sweden [12]. The average regional response rate was 42%, which is based on the denominators received from the chairpersons of 14 regions. Table 1 presents baseline characteristics.

**Table 1:** Baseline characteristics of the respondents and use of knowledge support

**Profession (n = 130)**	
Doctor	77 (59%)
Pharmacist	40 (31%)
Nurse	8 (6%)
Other	5 (4%)
**Experience working with pharmaceutical questions within the region (n = 131)**	
< 1 year	11 (8%)
1–5 years	43 (33%)
6–10 years	33 (25%)
> 10 years	44 (34%)
**Frequency of writing prescriptions to patients (n = 132)**	
No, never	54 (41%)
Yes, more rarely than once per week	17 (13%)
Yes, about once a week	11 (8%)
Yes, almost everyday	50 (38%)
**Role in the DTC** ^a^ **(n = 132)**	
Member of DTC	88 (67%)
Member of a therapy/expert group	81 (61%)
Employee at the Region’s pharmaceutical unit	42 (32%)
Other	18 (14%)
**Familiarity with the issue of pharmaceuticals in the environment (n = 132)**	
Not at all familiar	2 (1%)
Less familiar	43 (33%)
Somewhat familiar	75 (57%)
Very familiar	12 (9%)
Mean^b^ (n = 132)	2.7
**Importance of considering environmental impact in the formulary (n = 131)**	
Not at all important	1 (1%)
Less important	4 (3%)
Somewhat important	56 (42%)
Very important	68 (52%)
Do not know	2 (2%)
Mean^c^ (n = 129)	3.5
**Use of knowledge support** ^a^ **(n = 129)**	
Janusinfo	83 (64%)
Fass	64 (50%)
Other	26 (20%)
Do not know	34 (26%)

^a^Multiple options could be selected.

^b^The mean is calculated based on assigning the following values: Not at all familiar = 1; Less familiar = 2; Somewhat familiar = 3; Very familiar = 4. “Do not know” answers were excluded.

^c^The mean is calculated based on assigning the following values: Not at all important = 1; Less important = 2; Somewhat important = 3; Very important = 4. “Do not know” answers were excluded.

More than half of the respondents were doctors (59%); mode values were more than 10 years of experience working with pharmaceutical issues within the region (34%); prescribed pharmaceuticals for patients almost every day (38%); and members of their DTC (67%). More than half of the respondents were *somewhat familiar* with the topic of pharmaceuticals in the environment (57%). The mean value for familiarity (4-point Likert scale) was 2.7. Slightly above half thought it was *very important* to consider environmental impact in the formulary (52%). The mean value for the importance of considering environmental impact in the formulary (4-point Likert scale) was 3.5.

When recommending pharmaceuticals, given the choice to select multiple options, out of 129 answers, 83 respondents reported that the DTC used Janusinfo, 64 reported that the DTC used Fass, 26 reported the DTC using other sources, and 34 did not know which sources the DTC uses. Respondents mostly reported using scientific literature as an “other” source in addition to or instead of the knowledge supports.

### The knowledge supports

#### The use

Besides work with recommended pharmaceuticals, 52 respondents reported using environmental information in different areas of practice. For example, as information for education, communication, setting regional environmental goals and in the procurement of pharmaceuticals.

#### Usefulness

**Fig. 1:**
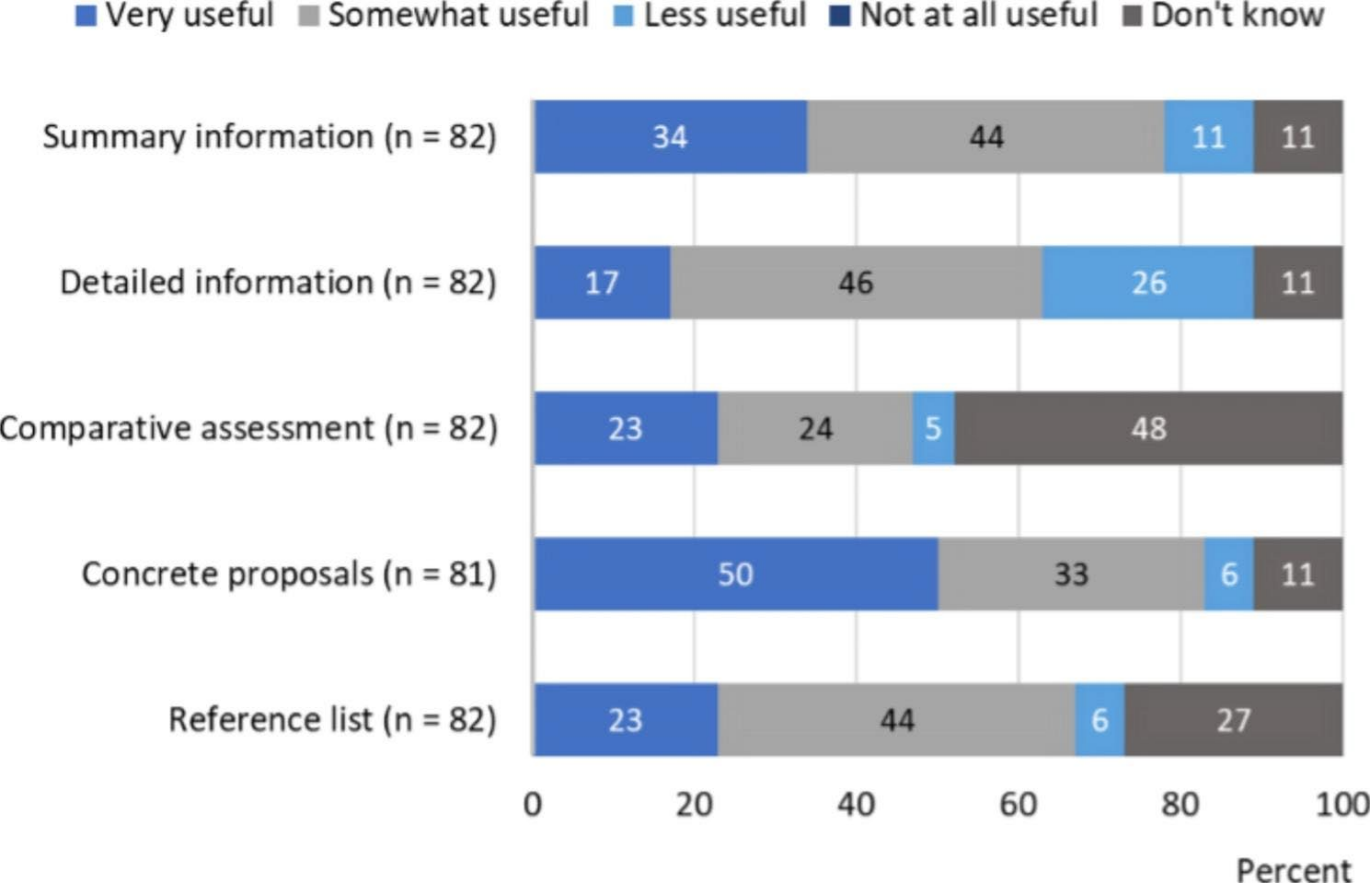
Usefulness of Janusinfo

**Fig. 2:**
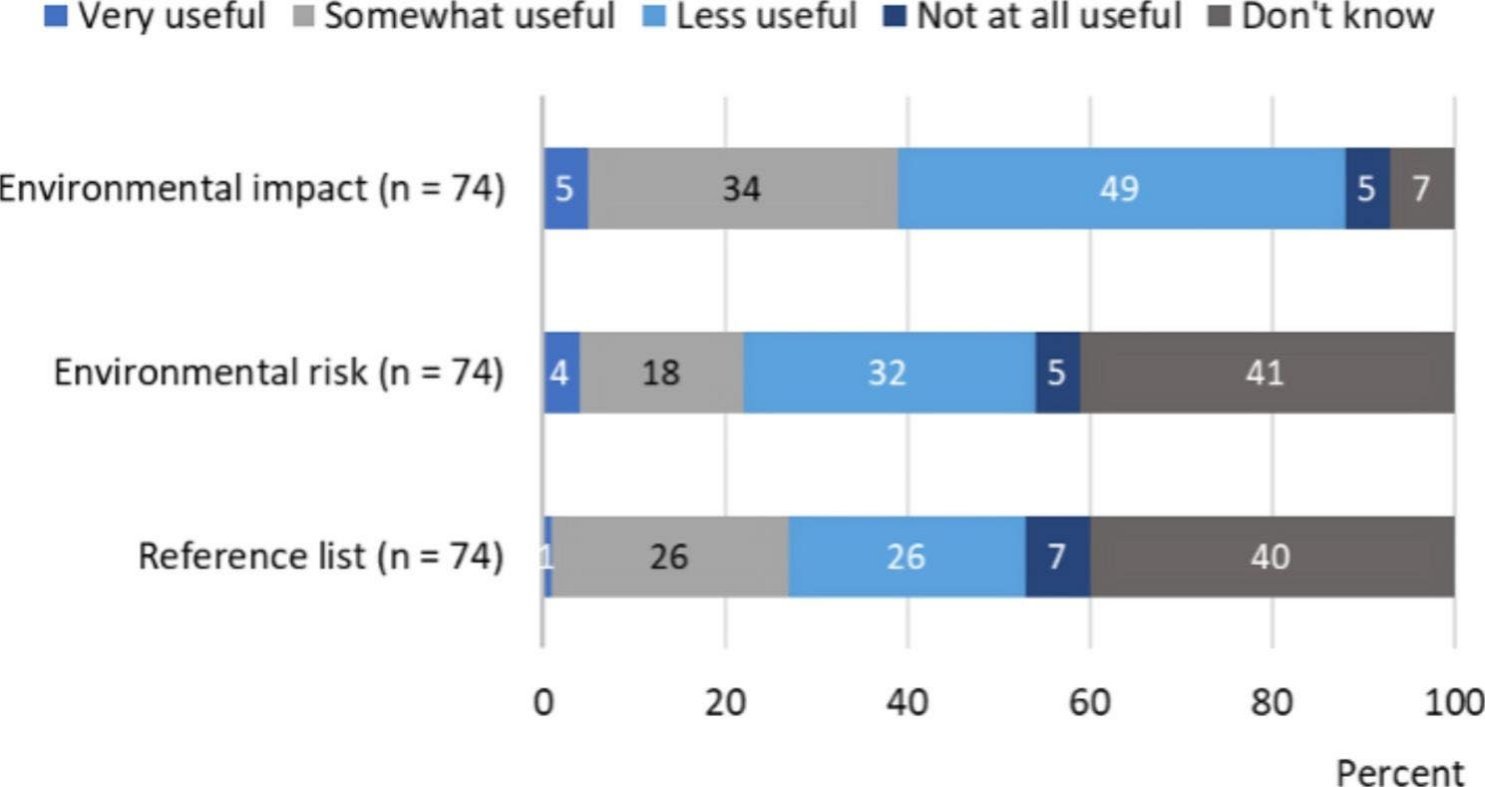
Usefulness of Fass

A total of 89 respondents had used the knowledge support for environmental information on pharmaceuticals available through either Janusinfo, Fass or both. Questions regarding the knowledge supports’ usefulness were asked to understand respondents’ attitudes on the environmental information. The findings are presented in Figs. 1 and 2. Data is presented in additional files [see Supplementary Material 2 and Supplementary Material 3]. Mode values for Janusinfo were *somewhat useful* (44%) for the summary, *somewhat useful* (46%) for the detailed information, *do not know* (48%) for the comparative assessments, *very useful* (50%) for the concrete proposals, and *somewhat useful* (44%) for the reference list. The mean values for the usefulness of Janusinfo (4-point Likert scale) were all above 3, except for the detailed information. The mean values greater than 3 indicate that respondents generally thought that the information was at least somewhat useful. The greatest mean value, 3.5 (n = 72), was associated with the concrete proposals.

Mode values for Fass were *less useful* (49%) for the environmental impact, and *do not know* for the environmental risk classification (41%) as well as the reference list (40%). The mean values for the usefulness of the information (4-point Likert scale) were all below 2.5. The greatest mean value, 2.4, was associated with the environmental impact and the reference list (n = 69 and n = 44, respectively). The mean values for Fass indicate that respondents generally thought the information was less useful.

#### Comprehensibility and helpfulness

Most respondents thought that the information on the knowledge supports was *somewhat easy* to comprehend, Janusinfo 59% vs. Fass 58%. A greater proportion of respondents stated that the information was *very easy* to comprehend on Janusinfo 24% compared to Fass 4%. Mean values for the comprehensibility of the information (4-point Likert scale) were 3.1 (n = 77) and 2.6 (n = 68) for Janusinfo and Fass respectively.

Respondents mostly reported that when using the knowledge support to make their formularies, the information was helpful to *a certain extent*, Janusinfo (58%) and Fass (69%). A greater proportion of respondents thought that the information on Janusinfo was helpful to *a large extent* compared to Fass (32% vs. 5%). No respondents thought that Janusinfo was *not at all helpful*, whereas 15% of respondents thought Fass was *not at all helpful*. Mean values for the helpfulness of the knowledge support (3-point Likert scale) were 2.4 (n = 74) and 1.9 (n = 65) for Janusinfo and Fass respectively. Additional data is presented in Supplementary Material 4.

#### Comments on Janusinfo and Fass through the open-ended questions

Tables 2 and 3 present results for the open-ended questions about what the respondents appreciated about each knowledge support and what suggestions they had for improvements. Highlights from what respondents appreciated about Janusinfo included that it is easily accessible, evidence-based, and a transparent source. Respondents also appreciated that the information is presented per API and that the knowledge support includes concrete proposals for how to mitigate especially environmentally harmful APIs. However, they would like to have information on more APIs, more comparative environmental assessments and text that is easier to understand.

**Table 2:** Specific questions about Janusinfo. “What do you think is good about the knowledge support “Pharmaceuticals and Environment” on Janusinfo?” Fifty-five out of 89 people answered the question for Janusinfo. Note that the number will be more than 55 as people may have specified several strengths. “What suggestions for improvements do you have for the knowledge support “Pharmaceuticals and Environment” on Janusinfo?” Thirty-two out of 89 people answered the question. Note that the number will be more than 32 as people may have specified several strengths

**What do you think is good about the knowledge support “Pharmaceuticals and Environment” on Janusinfo?**		
**Categories**	**# of comments**	**Selected examples**
Easily accessible, easy to understand and valuable summaries.	32	*“The information is presented per substance.”*
		*“Easily accessible and easy to understand.”*
Valuable, comprehensive, evidence-based, transparent source.	24	*“Concrete, practical and at the same time clearly evidence-based.”*
		*“Relevant information for both prescribers and the DTC. Credible source. High degree of transparency.”*
Environmentally harmful pharmaceuticals including concrete proposals on how to work to reduce emissions and environmental comparisons between similar substances.	17	*“The strength lies not least in the concrete proposals.”*
**What suggestions for improvements do you have for the knowledge support “Pharmaceuticals and Environment” on Janusinfo?**		
**Categories**	**# of comments**	**Selected examples**
Information on more substances including comparisons, update with new knowledge and concrete proposals.	11	*“More substances and comparisons between substances although it is difficult to find documentation on them, preferably more of the type, why is diclofenac much worse than other NSAIDs.”*
		*“More substances with alternatives.”*
Want more easily understood text, shorter text, and better search function.	10	*“Have information about what persistence, bioaccumulation and toxicity mean on each page so you can interpret the result more easily.”*
Other comments.	8	*“Sometimes very scarce information.”*
		*“Would have been nice with ONE clear system … and an explanatory comment on all medicines.”*
Do not know or question marks.	5	*n/a*
		*n/a*
Better marketing about the knowledge support “Pharmaceuticals and Environment”.	2	*“The biggest improvement proposal is probably that the knowledge that exists in this knowledge support would be more accessible to everyone in Swedish healthcare. As it is now, most prescribers in my own region unfortunately do not know that this data exists.”*

**Table 3:** Specific questions about Fass. “What do you think is good about the environmental information on Fass?” Thirty-one out of 89 people answered the question. “What suggestions for improvements do you have for Fass?” Thirty-five out of 89 people answered the question. Note that the number will be more than 35 as people may have specified several strengths

**What do you think is good about environmental information on Fass?**		
**Categories**	**# of comments**	**Selected examples**
Focus on the subject, comprehensive information and easily accessible.	19	*“That the issue is raised and made aware. Then the information needs to be developed.”*
		*“Available at all times.”*
No or limited benefit or do not know.	6	*n/a*
Easy to understand and concise.	4	*“The summary on environmental impact is very short and easy to understand, of course.”*
**What suggestions for improvements do you have for Fass?**		
**Categories**	**# of comments**	**Selected examples**
Environmental information for all medicinal products. Increased quality of information as well as increased harmonisation between different companies with the same API.	18	*“There should be environmental information for all medicinal products.”*
		*“More consistent information between companies, clearer overview.”*
Text that is easier to understand, and the environmental information needs to be more visible.	8	*“Hard to understand, so simpler words could be good.”*
		*“Quite difficult to relate to numbers and such. I feel that it requires that you are familiar with the area to understand.”*
Other comments.	7	*“Concrete recommendations are what the prescriber needs.”*
		*“It would have been great if there was a scale where all substances were placed in the same ATC code. That way it would be easier to orientate and quickly see which substance has the most/least impact on the environment.”*
Requests information on environmental aspects of manufacturing.	4	*“There is no information about the country of origin and possible transports or environmental aspects at the production site.”*
The source of information should be authorities and not the pharmaceutical companies.	3	*“Very fuzzy. In many cases, there is no nuanced picture when the pharmaceutical companies themselves make the assessment of a medicinal product’s environmental impact.”*
		*“Better if the information were producer independent.”*

Respondents appreciated that Fass presents environmental information for medicinal products, that it includes comprehensive information, and that it is easily accessible. Suggestions for improvement were that environmental information for all medicinal products should be provided and there should be harmonisation of information between different medicinal products with the same API. Environmental information about manufacturing was also requested. Some suggested that the information should come from the authorities instead of the pharmaceutical industry. Respondents also wanted a more easy-to-understand text.

### Challenges

The most perceived challenges in the work of reducing the environmental impact of pharmaceuticals concerned communication and availability of environmental information on pharmaceuticals for prescribers and the public (Table 4). Other challenges were associated with the lack of demands requiring the pharmaceutical industry to be more transparent about emissions during manufacturing and with a general desire for the industry to take greater environmental responsibility. Several believed that changes in the legislation are required for this to happen. Additionally, respondents stated that upgrading wastewater treatment plants for the treatment of pharmaceutical residues is needed. Respondents suggested that the healthcare system should work more preventatively, use more non-pharmacological treatments, and perform more medication reviews. Use of the information in healthcare practice was also a challenge and some respondents stated it was difficult to consider environmental aspects of pharmaceuticals in relation to other criteria such as efficacy and cost-effectiveness. Use of the information in practice can be limited by the fact that environmental information is lacking for many APIs.

**Table 4:** Comments about “What challenges do you see in the work of reducing environmental impact? “Ninety eight out of 132 people answered the question. Note that the number will be more than 98 as people may have specified several strengths

What challenges do you see in the work of reducing environmental impact?		
**Categories**	**# of comments**	**Selected examples**
Communicate knowledge and clear messages to prescribers/healthcare, that there is time and resources, information to the public, desirable with independent information.	32	*“It is important to give good examples of what happens to the environment when the medicine is used.”*
		*“Either it must be integrated into current recommendations, or you must have clear messages about what you can do.”*
Pharmaceutical industry: Impact from the pharmaceutical industry, desirable with increased transparency about pharmaceutical manufacturing, desirable to also be able to include environmental impact from pharmaceutical production and long logistics and manufacturing chains.	25	*“The knowledge about emissions, both of medicines, residual products, energy consumption, transport, etc. in the entire pharmaceutical chain must be investigated and reported. The work of making demands on pharmaceutical companies, and others who work with pharmaceuticals, to report and reduce its environmental impact (not only the impact of substances, but everything else around as well) must be built into all government work, e.g., approval processes, price discussions and procurements.”*
Tougher requirements for cost effectiveness, unclear how to consider environmental impact against efficacy and cost effectiveness, the environmental aspect in the second place and increased environmental requirements in procurement.	23	*“The increasingly stringent demand for cost-effectiveness and with our new expensive medicines, the environmental aspect may have a somewhat hidden place.”*
		*“Many medicines are needed, even though they are harmful to the environment.”*
Lack of knowledge about environmental impact, lack of environmentally better alternatives or information about treatments with lower impact.	16	*“Several medicines with an environmental impact cannot be replaced with more environmentally friendly alternatives. Environmental information may be missing.”*
		*“Lack of available environmental information complicates comparisons between pharmaceuticals.”*
The wastewater treatment plants need to be upgraded for the treatment of pharmaceutical residues.	15	*“It should also be obvious that pharmaceutical treatment is included as a mandatory requirement for wastewater treatment plants, as the majority of persistent substances are excreted in the environment.”*
		*“Wastewater treatment plants in Sweden, and other countries, must build in effective treatment for medicine residues.”*
Legislative changes are needed.	12	*“Changes in legislation are required for pharmaceutical companies to be forced to take greater environmental responsibility.”*
More interventions such as medication reviews and alternative treatments such as non-pharmacological treatments, preventive health work, medicines that are not used.	7	*“Another challenge, however, is society’s attitude towards medicines versus non-pharmacological treatment options. There is a sometimes-casual prescribing of medicines in cases where non-pharmacological alternatives are preferred. We also need to work more with medication reviews: all medicines must be followed up and stopped if they are not beneficial to the patient. In this way, we can reduce the number of medicines in nature.”*
		*“Healthcare should work more preventively and make more use of non-pharmacological treatments.”*
International issue.	3	*“Global problem.”*
		*“That international guidelines for the development of medicines cover environmental aspects.”*

## Discussion

We investigated the experiences with using knowledge supports for environmental information on pharmaceuticals among DTCs in Sweden. This survey, completed predominantly by DTC members, demonstrated that respondents generally appreciated the availability and accessibility of the information. However, few were very familiar with the issue demonstrating a need to present the information in a comprehensible way.

More respondents were familiar with Janusinfo compared to Fass, and the information on Janusinfo was assessed as more helpful as well as more comprehensible compared to Fass. Respondents especially appreciated the concrete proposals available for some APIs on Janusinfo. Increased availability of the concrete proposals for how to reduce especially harmful APIs was requested. Regarding Fass, respondents suggested environmental information for all medicinal products be presented. The respondents also thought harmonisation of information between different companies with the same API is necessary to improve Fass.

A challenge that was raised by many respondents was the lack of transparency from the pharmaceutical industry and the lack of responsibility to present environmental information about their medicinal products. Voluntary initiatives by pharmaceutical companies, such as environmental information on Fass, are welcome but have shortcomings [15, 20]. The respondents would also prefer to have the environmental information provided by the authorities as opposed to pharmaceutical companies. Environmental information from the authorities is advantageous given their objectivity and access to both public and confidential information [15]. This is in line with recommendations from the WHO stating that information independent from the pharmaceutical industry is a core intervention necessary to promote a more rational use of medicines [25]. It has been argued that based on legal requirements, it is necessary for the authorities to establish a public database based on APIs with substantiated information on the environmental risk assessments [26]. Some respondents also stated that providing environmental risk information, including from production, should be mandatory. National regulatory authorities know the producers of each medicinal product marketed in their country but claim they cannot share the information due to confidentiality. However, information on the manufacturing of pharmaceuticals has been made public in New Zealand [27, 28].

The lack of environmental information on pharmaceuticals is also a challenge for the DTCs wanting to incorporate environmental aspects into their regional formulary. This is a problem not least for medicinal products put on the market before the European Medicines Agency’s (EMA’s) guideline on environmental risk assessments for medicinal products for human use was implemented in 2006 [15, 29, 30]. Most APIs have inadequate or no ecotoxicological data [15, 31]. This makes it difficult to discriminate between different APIs [15, 31]. Even though respondents stated that they wanted information on more APIs on Janusinfo, Janusinfo is dependent on the availability of environmental information from EMA and Fass.

This study demonstrates that many respondents think that it is important to consider environmental aspects of pharmaceuticals, however, it is difficult for them to know how to incorporate the information in practice. Some respondents expressed difficulty with weighing the environment against other criteria, e.g., efficacy since medicines are important for human health despite environmental effects. To facilitate the DTCs’ work, respondents highlighted that legislation is needed. For example, environmental risk should be included in the overall benefit/risk assessment when a human medicinal product is considered for market authorisation.

Strengths of this study include that it covered almost all Swedish DTCs with respondents encompassing 18/21 Swedish regions and that it included both knowledge support systems, Janusinfo and Fass. Weaknesses of the study include the method of distribution and unclarity about present and past use. Some regions sent it out to more people while others were more restrictive. Thus, these regions in the first case may have a greater impact on the result through their greater representation and may reflect what is stated in regional instructions even though the survey was completed individually. Some people who answered the survey were not as well-versed with the subject matter, indicating the survey may have been forwarded to the wrong people. We also asked respondents to skip the questions about the respective knowledge supports if they were not using them. However, we included those who said they were using one knowledge support but still answered questions for both knowledge supports since they could have had previous experience using it.

Respondents wanted increased availability of concrete proposals for how to reduce especially harmful APIs for the knowledge support Janusinfo. However, APIs with similar medical effects can have the same environmental problems and the lack of environmental information makes providing concrete proposals difficult. Even if wastewater treatment plants are upgraded, as respondents noted as important, upstream mitigation strategies, initiatives taken to prevent the release of pharmaceutical residues into the aquatic environment, are still necessary [32, 33]. This includes the integration of environmental information in formularies on all levels, national and eventually international guidelines, medication reviews and non-pharmacological treatment [32–35]. The goal is to give the patient the best possible treatment with the least possible environmental impact.

## Conclusions

The DTCs in Sweden value the availability of public knowledge support for environmental information on pharmaceuticals. The knowledge supports gives them the opportunity to, among other things, consider environmental aspects of pharmaceuticals in their formularies. However, knowledge support for environmental information on pharmaceuticals needs to be provided with an approach that is both comprehensible and helpful for them. Challenges that were raised were lack of transparency from the pharmaceutical industry and lack of environmental information for many pharmaceuticals. This study can provide insights to those in other countries interested in considering environmental aspects in their formulary decision-making.

## Electronic supplementary material

Below is the link to the electronic supplementary material.

Supplementary Material 1

Supplementary Material 2

Supplementary Material 3

Supplementary Material 4

## Data Availability

The datasets are not publicly available, but anonymized data can be requested from the corresponding author.

## Footnotes

1 Sweden is divided into 21 regions: the three biggest (Skåne, Västra Götaland and Stockholm) have between 1.4 and 2.4 million inhabitants each. The smallest region (Gotland) has around 61,000 inhabitants and the other 17 regions have a population between 130,000 and 470,000 inhabitants [12].

## List of abbreviations

API: Active Pharmaceutical Ingredient
DTC: Drug and Therapeutics Committee
EBM: Evidence-Based Medicine
EMA: European Medicines Agency
iPiE: Intelligent Assessment of Pharmaceuticals in the Environment
LIF: The trade association for the research-based pharmaceutical industry in Sweden
WHO: World Health Organization
