# Supplementary material for: Knowledge support for environmental information on pharmaceuticals: experiences among Swedish Drug and Therapeutics Committees
Source: BMC Health Serv Res. 2023 Jun 12;23:618. doi: 10.1186/s12913-023-09646-7 (PMC10259041; doi:10.1186/s12913-023-09646-7)
Supplement: Supplementary file 1 — Supplementary Material 1 [file 12913_2023_9646_MOESM1_ESM.docx]

# Supplementary Material 1. Survey on Drug and Therapeutics Committees’ use of knowledge support for environmental information on pharmaceuticals

## General Questions

1. Which region do you work in? _______________

2. Which role(s) do you have in the region’s pharmaceutical work? (Select all that apply)

Member of the Drug and Therapeutics Committee

Therapy/expert group member

Employee at the region’s pharmaceutical unit

Other, specify which: _________________________

3. Professional background

Pharmacist

Doctor

Nurse

Other, specify which: _________________________

4. How long have you worked with pharmaceutical questions in the region?

Less than one year

1‒5 years

6‒10 years

More than 10 years

5. Do you prescribe pharmaceuticals to patients?

Yes, almost every day

Yes, approximately once a week

Yes, more rarely than once a week

No, never

6. How familiar are you with the issue of pharmaceuticals in the environment?

| Not at all familiar | Less familiar | Somewhat  familiar | Very  familiar | Don’t  know |
| --- | --- | --- | --- | --- |
|  |  |  |  |  |

7. How important do you consider it is to include environmental aspects in the selection of pharmaceuticals for the recommendation’s list?

| Not at all  Important | Less  Important | Somewhat important | Very  important | Don’t  know |
| --- | --- | --- | --- | --- |
|  |  |  |  |  |

8. How does your Drug and Therapeutics Committee consider environmental aspects in pharmaceutical recommendations or in other information about pharmaceuticals?

__________________________________________________________________________________________________________________________________________________________________________

__________________________________________________________________________________________________________________________________________________________________________

## Knowledge supports for environmental information on pharmaceuticals

## 9. Which knowledge support for environmental information on pharmaceuticals does the Drug and Therapeutic’s Committee use when recommending pharmaceuticals?

Janusinfo.se

Fass.se

Other, _________________________________________________________________________

Don’t know

*If not Janusinfo.se, skip questions 10–14

* If not Fass.se, skip questions 15–18

* If don't know, skip questions 10‒18

## Supplementary Material 1. Environmental information in the knowledge support “Pharmaceuticals and Environment” on Janusinfo.se

1. When you use the knowledge support “Pharmaceuticals and Environment” on Janusinfo.se for pharmaceutical substances, how useful do you consider the following information to be?

|  | Not at all  useful | Less  useful | Somewhat useful | | Very useful | Don’t  know | |  |
| --- | --- | --- | --- | --- | --- | --- | --- | --- |
| Initial information on persistence, bioaccumulation, toxicity, and risk? |  |  |  | |  |  | |  |
| Detailed information from the various sources, Fass and EMA? |  |  |  | |  |  | |  |
| Environmental comparison for pharmaceutical substances with similar effect found in the reports from Goodpoint? |  |  |  | |  |  | |  |
| Concrete action proposals for certain pharmaceuticals, e.g., sertaline, diclofenac, ethinyl estradiol |  |  |  | |  |  | |  |
| The reference list with web links? |  |  |  |  | |  |  | |

1. When you use the knowledge support “Pharmaceuticals and Environment” on Janusinfo.se, how easy is it to understand the texts for pharmaceutical substances?

| Very  difficult | | Somewhat difficult | | Somewhat easy | | Very  easy | Don’t know | |
| --- | --- | --- | --- | --- | --- | --- | --- | --- |
|  |  | |  | |  | |  |  |

1. When you use the knowledge support “Pharmaceuticals and Environment” on Janusinfo.se, is the information helpful in the work with recommending pharmaceuticals?

| Not at all | To a certain  extent | To a large  extent | Don’t know |
| --- | --- | --- | --- |
|  |  |  |  |

1. What do you think is good about the knowledge support “Pharmaceuticals and Environment” on Janusinfo.se?

__________________________________________________________________________________________________________________________________________________________________________

__________________________________________________________________________________________________________________________________________________________________________

1. What suggestions for improvement do you have for the knowledge support “Pharmaceuticals and Environment” on Janusinfo.se?

__________________________________________________________________________________________________________________________________________________________________________

__________________________________________________________________________________________________________________________________________________________________________

## Environmental information on Fass.se

1. When you use the environmental information on Fass.se, how useful do you consider the following information to be?

|  | Not at all useful | Less  useful | Somewhat  useful | Very  useful | Don’t  know |
| --- | --- | --- | --- | --- | --- |
| Environmental impact (environmental risk,  degradation, bioaccumulation?) |  |  |  |  |  |
| Environmental classification  (PEC/PNEC)? |  |  |  |  |  |
| The reference  list? |  |  |  |  |  |

1. When you use the environmental information on Fass.se, how easy is it to understand the texts for medicinal products?

| Very  difficult | Somewhat difficult | Somewhat easy | Very  easy | Don’t  know |
| --- | --- | --- | --- | --- |
|  |  |  |  |  |

1. When you use the environmental information on Fass.se, is the information helpful in the work with recommending pharmaceuticals?

| Not at all | To a certain extent | To a large  extent | Don’t  know |
| --- | --- | --- | --- |
|  |  |  |  |

1. What do you think is good about the environmental information on Fass.se?

__________________________________________________________________________________________________________________________________________________________________________

__________________________________________________________________________________________________________________________________________________________________________

1. What suggestions for improvement do you have for the environmental information on Fass.se?

__________________________________________________________________________________________________________________________________________________________________________

__________________________________________________________________________________________________________________________________________________________________________

## Other Questions

20. Does the region use information on pharmaceuticals’ impact on the environment in ways other than the work with Drug and Therapeutics Committee’s pharmaceutical recommendations?

Yes, how? ____________________________________________________________

No

Don’t know

21. What challenges do you see in the work to reduce the environmental impact of pharmaceuticals?

__________________________________________________________________________________________________________________________________________________________________________

__________________________________________________________________________________________________________________________________________________________________________
