# Supplementary material for: Knowledge support for environmental information on pharmaceuticals: experiences among Swedish Drug and Therapeutics Committees
Source: BMC Health Serv Res. 2023 Jun 12;23:618. doi: 10.1186/s12913-023-09646-7 (PMC10259041; doi:10.1186/s12913-023-09646-7)
Supplement: Supplementary file 2 — Supplementary Material 2 [file 12913_2023_9646_MOESM2_ESM.docx]

**Supplementary Material 2.** Usefulness of Janusinfo.

|  | **Summary information**  (n = 82)  no. (%) | **Detailed information**  (n = 82)  no. (%) | **Comparative assessments**  (n = 82)  no. (%) | **Concrete proposals**  (n = 81)  no. (%) | **Reference  list**  (n = 82)  no. (%) |
| --- | --- | --- | --- | --- | --- |
| Not at all useful | 0 (0) | 0 (0) | 0 (0) | 0 (0) | 0 (0) |
| Less useful | 9 (11) | 21 (26) | 4 (5) | 5 (6) | 5 (6) |
| Somewhat useful | 36 (44) | 38 (46) | 20 (24) | 27 (33) | 36 (44) |
| Very useful | 28 (34) | 14 (17) | 19 (23) | 40 (50) | 19 (23) |
| Don’t know | 9 (11) | 9 (11) | 39 (48) | 9 (11) | 22 (27) |
| Mean^[[1]](#footnote-1)^ | 3.3 (n=73) | 2.9 (n=73) | 3.3 (n=43) | 3.5 (n=72) | 3.2 (n=60) |

1. The mean is calculated based on assigning the following values: Not at all useful = 1; Less useful = 2; Somewhat important = 3; Very important = 4. “Don’t know” answers were excluded. [↑](#footnote-ref-1)
