# Supplementary material for: Knowledge support for environmental information on pharmaceuticals: experiences among Swedish Drug and Therapeutics Committees
Source: BMC Health Serv Res. 2023 Jun 12;23:618. doi: 10.1186/s12913-023-09646-7 (PMC10259041; doi:10.1186/s12913-023-09646-7)
Supplement: Supplementary file 3 — Supplementary Material 3 [file 12913_2023_9646_MOESM3_ESM.docx]

**Supplementary Material 3.** Usefulness of Fass.

|  | **Environmental impact**  (n = 74)  no. (%) | **Environmental risk classification**  (n = 74)  no. (%) | **Reference  list**  (n = 74) no. (%) |
| --- | --- | --- | --- |
| Not at all useful | 4 (5) | 4 (5) | 5 (7) |
| Less useful | 36 (49) | 24 (32) | 19 (26) |
| Somewhat useful | 25 (34) | 13 (18) | 19 (26) |
| Very useful | 4 (5) | 3 (4) | 1 (1) |
| Don’t know | 5 (7) | 30 (41) | 30 (40) |
| Mean^[[1]](#footnote-1)^ | 2.4 (n = 69) | 2.3 (n = 44) | 2.4 (n = 44) |

1. The mean is calculated based on assigning the following values: Not at all useful = 1; Less useful = 2; Somewhat important = 3; Very important = 4. “Don’t know” answers were excluded. [↑](#footnote-ref-1)
