# Supplementary material for: Knowledge support for environmental information on pharmaceuticals: experiences among Swedish Drug and Therapeutics Committees
Source: BMC Health Serv Res. 2023 Jun 12;23:618. doi: 10.1186/s12913-023-09646-7 (PMC10259041; doi:10.1186/s12913-023-09646-7)
Supplement: Supplementary file 4 — Supplementary Material 4 [file 12913_2023_9646_MOESM4_ESM.docx]

**Supplementary Material 4.** Comprehensibility and helpfulness of knowledge support.

| **Comprehensibility of knowledge support** | | |
| --- | --- | --- |
|  | **Janusinfo**  (n = 82)  no. (%) | **Fass** (n = 73)  no. (%) |
| Very difficult | 0 (0) | 4 (5) |
| Somewhat difficult | 9 (11) | 19 (26) |
| Somewhat easy | 48 (59) | 42 (58) |
| Very easy | 20 (24) | 3 (4) |
| Don’t know | 5 (6) | 5 (7) |
| Mean^[[1]](#footnote-1)^ | 3.1 (n = 77) | 2.6 (n = 68) |
| **Helpfulness of knowledge support** | | |
|  | **Janusinfo**  (n = 82)  no. (%) | **Fass**  (n = 73)  no. (%) |
| Not at all | 0 (0) | 11 (15) |
| To a certain extent | 48 (58) | 50 (69) |
| To a large extent | 26 (32) | 4 (5) |
| Don’t know | 8 (10) | 8 (11) |
| Mean^[[2]](#footnote-2)^ | 2.4 (n = 74) | 1.9 (n = 65) |

1. The mean is calculated based on assigning the following values: Very difficult = 1; Somewhat difficult = 2; Somewhat easy = 3; Very easy = 4. “Don’t know” answers were excluded. [↑](#footnote-ref-1)
2. The mean is calculated based on assigning the following values: Not at all = 1; To a certain extent = 2;
    To a large extent = 3. “Don’t know” answers were excluded. [↑](#footnote-ref-2)
